# The impact of ward noise management and comprehensive nursing intervention on cognitive function, psychological health, and prognosis of post-stroke patients: a randomized controlled trial

**DOI:** 10.3389/fneur.2026.1778278

**Published:** 2026-03-03

**Authors:** Yuan Deng, Yanmin Liu, Jie Liu, Yufei Sang, Fang Wang

**Affiliations:** 1 Department of Nursing, Yueyang Vocational Technical College, Yueyang, China; 2 Medical Services Department, 923th Hospital of Joint Logistic Support Force of PLA, Nanning, China; 3 Department of Health Management College, Xianning Vocational Technical College, Xianning, China

**Keywords:** cognition, mental health, noise, prognosis, public health nursing, rehabilitation, stroke

## Abstract

**Objective:**

This study aimed to investigate the impact of ward noise management combined with comprehensive nursing intervention on cognitive function, psychological health, and prognosis in post-stroke patients.

**Methods:**

A total of 144 stroke patients admitted between January 2022 and December 2023 were randomized into a control group and an experimental group, with 72 patients in each group. The control group received conventional care, while the experimental group received systematic ward noise management (including noise control of medical equipment, medical staff, other patients in the same ward, and visiting family members) in addition to comprehensive nursing intervention (encompassing holistic assessment, individualized care plans, multidisciplinary collaboration, patient education, and continuous monitoring). Cognitive function, psychological status, motor function, quality of life, and complication rates were assessed using the Montreal Cognitive Assessment (MoCA), Mini-Mental State Examination (MMSE), Fugl-Meyer Assessment (FMA), Hamilton Depression Rating Scale (HAMD), Hamilton Anxiety Rating Scale (HAMA), Stroke-specific Quality of Life Scale (SS-QOL), and complication records.

**Results:**

The results showed that after the intervention, the experimental group demonstrated significantly greater cognitive recovery than the control group, with large effect sizes for MoCA (Cohen’s *d* = 0.75, 95% CI: 0.45–1.05) and MMSE (*d* = 0.80, 95% CI: 0.50–1.10). Motor function (FMA) improved more substantially in the experimental group, yielding a mean difference (MD) of 13.71 (95% CI: 9.94–17.48). Similarly, psychological well-being improved significantly, with MDs of −5.03 for HAMD (95% CI: −6.25 to −3.81) and −2.82 for HAMA (95% CI: −3.80 to −1.84). Quality of life (SS-QOL) also showed a moderate-to-large increase (MD = 13.23, 95% CI: 3.83–22.63). Furthermore, the experimental group had significantly lower odds of complications (OR = 0.35, 95% CI: 0.18–0.70; *p* = 0.021) compared to the control group.

**Conclusion:**

In conclusion, the results suggest that the integration of ward noise management with comprehensive nursing intervention may contribute to improvements in cognitive function, motor recovery, psychological health, quality of life, and clinical prognosis in post-stroke patients. This combined approach offers a promising strategy for optimizing rehabilitation outcomes and reducing complications. Further multicenter studies with larger sample sizes are recommended to validate these findings and refine intervention protocols.

## 1 Introduction

Stroke, a prevalent and serious neurological disorder, has profound physical and psychological consequences for patients and their families. During post-stroke rehabilitation, both the ward environment and nursing practices play crucial roles, as they are closely linked to patient prognosis, cognitive recovery, and psychological well-being (1). A quiet, clean, well-lit, and properly ventilated ward helps patients relax physically and cooperate more fully with treatment, thereby supporting recovery (2). Moreover, post-stroke patients require personalized care plans to address their diverse rehabilitation needs (3), such as physical, occupational, and speech therapy. Negative emotions like depression and anxiety represent another challenge in stroke recovery, making psychological interventions essential for improving patients’ mental health (4).

Ward noise poses a significant challenge to stroke rehabilitation. High decibel levels can impair sleep quality and increase anxiety, potentially worsening the clinical condition of stroke patients (5). Additionally, noise in the ward is associated with physiological stress responses, including increased heart rate and elevated blood pressure, which may hinder recovery (6). Research indicates that good sleep quality benefits both neural repair and functional recovery after stroke (7). Therefore, effective noise management is vital—measures may include using sound-absorbing materials, optimizing ward layout to reduce noise exposure, and training staff in noise-reduction practices.

Comprehensive nursing intervention represents a holistic approach that integrates physical and psychological care (8). For post-stroke patients, such interventions can encompass multiple aspects of treatment: physical rehabilitation, psychological support, medication management, and social support (9). This multidimensional strategy aims to promote complete physical and mental recovery. Previous studies have shown that comprehensive nursing intervention significantly improves the quality of life of stroke patients compared to partial or fragmented care approaches (10).

Currently, there is limited research on the integration of ward noise management with comprehensive nursing intervention in stroke rehabilitation. This study therefore seeks to explore the combined effect of structured noise control and holistic nursing on post-stroke recovery, with the goal of supporting clinical application.

## 2 Materials and methods

### 2.1 Baseline profiles

The study flow followed the CONSORT guidelines. The sample size was determined based on a power analysis using G*Power software. To detect a medium effect size (Cohen’s *d* = 0.50) for the primary outcome with a two-tailed alpha of 0.05 and a power of 0.80, a minimum of 64 patients per group was required. Accounting for a potential 10% dropout rate, we aimed to recruit approximately 144 patients (72 per group). Initially, 158 patients were screened; 14 were excluded (8 did not meet inclusion criteria, 6 declined). Thus, 144 patients were randomized 1:1 into the control (*n* = 72) and experimental (*n* = 72) groups. The randomization sequence was generated by an independent statistician using a computer-generated random number table. A block randomization method was employed with a fixed block size of four to ensure a balanced 1:1 allocation ratio between the experimental and control groups throughout the recruitment period. To ensure allocation concealment, the assignments were placed in sequentially numbered, opaque, sealed envelopes, which were opened by a research assistant only after the participants’ baseline assessments were completed and eligibility was confirmed.

**Figure 1:**
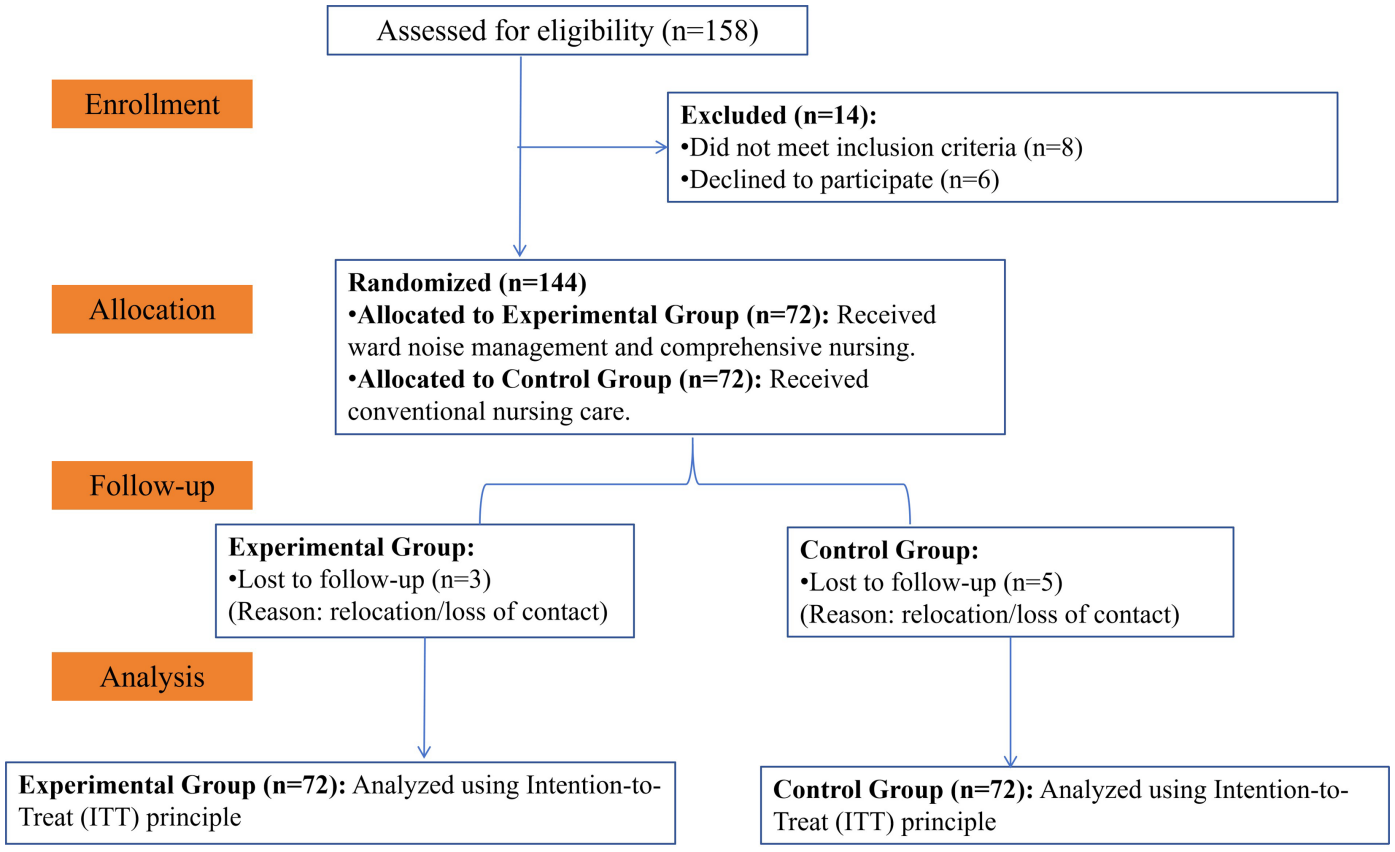
CONSORT flow diagram.

As shown in Figure 1 (CONSORT Flow Diagram), 5 patients in the control group and 3 in the experimental group were lost to follow-up during the one-year period, but all 144 randomized patients were retained for the final analysis according to the Intention-to-Treat (ITT) principle. The study received approval from the Ethics Committee of 923th Hospital of Joint Logistic Support Force of PLA (Approval No. 20220213 and was conducted in accordance with the approved protocol. All participants received their allocated intervention. No changes were made to the primary or secondary trial outcomes after the trial commenced. All pre-specified endpoints, including cognitive function, psychological status, and complication rates, were assessed according to the original study protocol without modification to ensure the integrity of the findings.

### 2.2 Inclusion and exclusion criteria

#### 2.2.1 Inclusion criteria

1. Age between 18 and 80 years.
2. Brain CT and MRI scans confirming the diagnosis of stroke (11).
3. First-time occurrence with onset within 14 days.
4. Hemiparesis grade for all participating hemi paretic patients within levels 0-III, and their vital signs must remain stable.
5. No severe cardiovascular diseases, liver or kidney failure, cancer, or other major health issues.
6. All participating patients and their families voluntarily agreed to participate in the study and signed the informed consent form.

#### 2.2.2 Exclusion criteria

1. Patients with comorbid conditions such as immune system abnormalities or coagulation disorders. These may complicate stroke recovery, interfere with treatment efficacy, increase the risk of infections or other complications during the study, and thereby obscure a reliable assessment of the intervention’s effects.
2. Patients with concurrent traumatic brain injury, hemorrhagic stroke, cerebral vascular malformation, epilepsy, or other significant brain-related diseases. Such conditions could independently influence cognitive, motor, and psychological outcomes, confounding the interpretation of the stroke-specific intervention under study.
3. Patients who have undergone arterial puncture within the preceding 7 days. Recent arterial procedures carry risks of bleeding or infection that may affect recovery, limit full participation, and compromise the validity of outcome measures.
4. Patients with contraindications to surgery. Underlying health issues that preclude surgical intervention may also impair the ability to engage in or benefit from rehabilitation, potentially affecting both study results and patient safety.
5. Patients with additional brain injuries, such as cerebral infarction. Coexisting cerebral lesions may produce deficits not attributable to the index stroke, making it difficult to isolate the effects of the rehabilitation intervention.
6. Patients with psychiatric disorders, as well as pregnant or lactating women. Psychiatric conditions may interfere with psychological evaluations and rehabilitation adherence, thereby skewing outcomes. Pregnancy and lactation are excluded to avoid potential risks to the fetus or infant from study interventions or concomitant medications, ensuring the safety of both mother and child.

### 2.3 Methods

The control group received standard conventional care, which involved routine medical care, without specific attention to environmental noise management or integrated, holistic care. The experimental group received an amalgamated intervention of ward noise management and comprehensive nursing. To ensure reproducibility, a structured intervention protocol was implemented as detailed in Supplementary Table S2 (Intervention Protocol). These interventions were standardized through detailed protocols that specified the frequency, duration, and intensity of the actions. These interventions were standardized through detailed protocols that specified the frequency, duration, and intensity of the actions. Additionally, the adherence to these protocols was monitored through weekly audits and ongoing nurse education.

#### 2.3.1 Noise management

##### 2.3.1.1 Management of medical equipment

Medical equipment was maintained regularly to minimize operational noise. This included using low-noise settings on mobile devices, selecting quiet inkjet printers, and prohibiting printer use at night. Printers were placed away from patient wards. Ward inspections were conducted frequently during the day, while nursing activity during night-time infusions was minimized. Paging system volumes were lowered, and monitoring devices were positioned away from the patient’s head, with alarm levels adjusted according to individual clinical status. All interventions were applied consistently during both daytime and nighttime hours, with daily monitoring to ensure compliance. Maintenance and noise-control measures were logged daily to ensure protocol adherence.

##### 2.3.1.2 Management of medical staff

Staff were instructed to maintain a quiet environment by speaking softly, handling equipment and doors gently, and moving carefully. These practices were enforced across all shifts, with particular emphasis on the night shift (10 p.m.–6 a.m.) to reduce disturbances. Training was provided initially and reinforced through monthly refreshers on noise-reduction protocols. Work schedules were organized to consolidate activities such as teaching rounds in designated areas. During ward rounds, students and staff were advised not to discuss other patients’ conditions in front of the participant, and verbal interactions during night inspections were kept to a minimum.

Environmental modifications were also implemented, including the use of soundproofing materials and soft-closing doors. Wards were situated away from streets and boiler rooms, and nighttime construction within the hospital premises was prohibited. These integrated measures aimed to create a calmer environment conducive to patient rest and recovery.

##### 2.3.1.3 Noise management of other patients in the same ward

Noise from other patients—such as groaning, shouting, or snoring due to pain, agitation, or illness—was actively addressed. Psychological support was strengthened to help patients cope with stress. When unusual sounds occurred, explanations and reassurance were offered promptly. For patients exhibiting heightened anxiety, distraction techniques were employed. Close monitoring ensured timely relief of discomfort, especially pain, to prevent noise disturbance.

A dedicated nursing supervisor was assigned to monitor the consistent application of all noise-management and comprehensive care protocols, thereby ensuring intervention fidelity. Specifically, daily checklists were used to document compliance with noise-reduction tasks (e.g., equipment volume checks), and weekly fidelity audits were conducted by the lead research team to ensure care plans remained individualized and active. Daily checklists were used to document compliance, and weekly fidelity checks were conducted by the research team. In cases where patients are experiencing pain or restlessness, the administration of appropriate analgesics or sedatives should be carried out under the guidance of the physician. Moreover, during the critical process of resuscitating severely ill patients, employing screens for isolation and minimizing the presence of bystanders helps mitigate the impact of noise on other patients.

##### 2.3.1.4 Noise from patients’ family members

Loud conversations, phone calls, and other noises from family members, attendants, and visitors can cause significant distress to patients. Managing this source of noise is therefore a priority in nursing administration. Upon admission, families are informed about visitation policies and access control, with emphasis placed on the negative impact of excessive noise. Strict measures are enforced to prevent noise during visiting hours. Limiting the number of companions during routine family gatherings is also stressed. In cases of non-compliance, family members or caregivers are politely asked to leave the ward.

#### 2.3.2 Comprehensive nursing

Comprehensive nursing is a holistic and individualized care approach designed to meet patients’ health needs. Its main components include:

1. Assessment: a thorough physical and psychological evaluation is conducted to understand the patient’s health status, medical history, lifestyle, and specific needs.
2. Individualized care plan: based on the assessment, a tailored plan is developed, covering treatment goals, medication, pain management, and nutrition.
3. Nursing team: care is delivered by a multidisciplinary team—including nurses, physicians, physical therapists, and social workers—working collaboratively.
4. Education and support: patients and families receive education on disease management, prevention, and rehabilitation to improve health literacy and self-management skills.
5. Treatment and rehabilitation: the plan emphasizes active treatment and rehabilitation, such as medication management, physical therapy, and exercise, to enhance quality of life.
6. Continuous monitoring: the patient’s progress is regularly monitored, and the care plan is adjusted as needed.
7. Communication and coordination: effective communication and coordination within the team ensure integrated and consistent care delivery.

#### 2.3.3 Conventional care

Conventional care refers to the standard, routine practices used in clinical settings to address patients’ basic needs and maintain their health and comfort. It generally includes:

1. Basic activities of daily living (ADLs): assisting with bathing, dressing, feeding, and hydration.
2. Vital signs monitoring: regularly checking temperature, pulse, respiration, and blood pressure.
3. Medication management: administering prescribed drugs, documenting their use, and monitoring effects.
4. Wound care: dressing and managing wounds to promote healing and prevent infection.
5. Communication with patients and families: providing illness and treatment information, answering questions, and offering emotional support.
6. Safety measures: implementing fall prevention, pressure ulcer prevention, and infection control.
7. Pain management: assessing and alleviating pain to ensure patient comfort.
8. Dietary management: ensuring adequate nutrition according to the patient’s needs.
9. Mobility assistance: helping with bed transfers and repositioning to prevent musculoskeletal complications.

Detailed intervention protocols and fidelity monitoring procedures are summarized in Supplementary Table S2.

### 2.4 Assessment indicators

#### 2.4.1 Primary endpoints

##### 2.4.1.1 Cognitive function

Cognitive function was assessed using two standard screening tools: the Montreal Cognitive Assessment (MoCA) and the Mini-Mental State Examination (MMSE). Both scales are widely employed to evaluate cognitive status and screen for conditions such as mild cognitive impairment and dementia (12). The MoCA covers multiple cognitive domains, including memory, executive functions, attention, language, visuospatial abilities, and abstract thinking. Tasks include word recall, drawing geometric figures, connecting numbered dots, clock drawing, and animal naming. Total scores range from 0 to 30, with scores below 26 suggesting possible cognitive impairment. The MMSE consists of a series of questions and tasks designed to assess overall mental status. Items include orientation to time and place, word repetition, serial subtraction, clock drawing, and answering simple questions. Scores also range from 0 to 30, and a score below 24 is generally indicative of cognitive concerns.

Assessments were performed at baseline (upon admission) and at the completion of the 4-week intensive intervention period to evaluate immediate outcomes. To minimize detection bias, all clinical assessments (including MoCA, MMSE, FMA, HAMD, HAMA, and SS-QOL) were conducted by trained outcome assessors who were masked to the treatment allocation. The blinding of assessors was maintained throughout the study and during the initial data entry process. Specifically, scores of 21–26 indicate mild intellectual impairment, 10–20 indicate moderate cognitive impairment, and 0–9 indicate severe cognitive impairment.

##### 2.4.1.2 Psychological status

Psychological status was measured using the Hamilton Depression Rating Scale (HAMD) and the Hamilton Anxiety Rating Scale (HAMA), with higher scores indicating more severe symptoms (13). The HAMD consists of 17 items rated on a 5-point scale (0–4), yielding a total score ranging from 0 to 68 (Cronbach’s *α* = 0.89, 95% CI 0.86–0.92 in this study). The HAMA comprises 14 items similarly scored on a 5-point scale, with a total score ranging from 0 to 56. Both assessments were administered at baseline (upon admission) and 4 weeks after the intervention.

##### 2.4.1.3 Motor function

Motor function was assessed using the Fugl-Meyer Assessment (FMA), a commonly used clinical tool to evaluate motor recovery in stroke patients (14). The FMA evaluates motor function of the upper limb (shoulder, elbow, wrist, and hand) and lower limb (hip, knee, and ankle), as well as sensory function (light touch, pain, and proprioception). Specific tasks such as arm lifting, knee flexion, and standing are performed and scored. The total motor score ranges from 0 to 100 (66 points for the upper limb, 34 points for the lower limb), with higher scores indicating better motor recovery. Assessments were conducted at baseline and 4 weeks post-intervention.

#### 2.4.2 Secondary endpoints

##### 2.4.2.1 Quality of life

Quality of life was assessed using the Stroke-specific Quality of Life Scale (SS-QOL), a tool designed to evaluate the physical, psychological, and social well-being of stroke patients and to measure treatment effects and rehabilitation progress (15). The scale comprises 49 items across 12 domains, including physical function, cognition, social relations, emotional status, mobility, and economic situation. Each item is scored on a 5-point scale, yielding a total score ranging from 0 to 245, with higher scores reflecting better quality of life. Assessments were conducted at baseline (upon admission) and 4 weeks after the intervention.

##### 2.4.2.2 Prognostic assessment

Rehabilitation prognosis was evaluated based on the incidence rate of complications. After the 4-week intervention, a structured one-year follow-up regimen was implemented via monthly outpatient visits and telephonic interviews to track long-term recovery. The incidence of complications within this year was recorded. To ensure diagnostic accuracy, all reported complications were validated by two independent neurologists masked to the group assignment, based on clinical symptoms and supplementary diagnostic tests (e.g., imaging for cerebral edema, laboratory cultures for infections). Major complications monitored included cerebral edema, infection, electrolyte imbalance, fever, seizures, and pressure ulcers. The complication incidence rate is calculated as: Number of individuals with complications/Total number of individuals × 100%.

### 2.5 Statistical methods

All statistical analyses were performed using SPSS software (version 23.0), and figures were generated using GraphPad Prism (version 9.0). Continuous data were presented as mean ± standard deviation (SD) and were compared between groups using independent samples t-tests. Categorical data were expressed as number (percentage, %) and analyzed using the *χ*^2^ test. To account for baseline differences between groups due to the repeated-measures design (pre and post-intervention), an analysis of covariance (ANCOVA) with baseline scores as the covariate was employed. Statistical significance was set at a two-sided *p*-value < 0.05.

For continuous outcomes, the effect size was reported as Cohen’s *d* alongside the mean difference (MD) with its 95% confidence interval (CI). For the incidence of complications, the odds ratio (OR) with its 95% CI was calculated to estimate the relative risk.

Data were analyzed according to the intention-to-treat (ITT) principle. Missing outcome data during the follow-up period were assumed to be missing at random (MAR) and were handled using multiple imputation with predictive mean matching (PMM). Sensitivity analyses were conducted to compare the results of the ITT analysis with those from the per-protocol analysis.

## 3 Results

### 3.1 Baseline profiles

A total of 144 patients were included in the ITT analysis. The use of Multiple Imputation for the 8 cases lost to follow-up ensured that the prognostic results remained robust. Sensitivity analysis showed no significant difference between the ITT-based findings and the per-protocol analysis, confirming the reliability of the intervention’s impact on long-term prognosis.

In the control group, there were 44 male patients and 28 female patients, aged 45 to 85 years with an average age of 63.78 ± 5.43 years. Among these patients, 36 had ischemic stroke, and 36 had hemorrhagic stroke. There were 38 patients with left-sided hemiparesis and 34 with right-sided hemiparesis. The experimental group consisted of 42 male patients and 30 female patients, aged 45 to 87 years with an average age of 65.53 ± 5.78 years. Among these patients, 35 had ischemic stroke, and 37 had hemorrhagic stroke. There were 41 patients with left-sided hemiparesis and 31 with right-sided hemiparesis. The two groups were well-balanced in terms of baseline profiles (*p* > 0.05), as shown in Table 1.

**Table 1:** Baseline profiles of patients.

Groups	*n*	Gender		Mean age	Ischemic stroke	Hemorrhagic stroke	Left-sided hemiparesis	Right-sided hemiparesis
		Male	Female					
Control	72	44	28	63.78 ± 5.43	36	36	38	34
Experimental	72	38	34	65.53 ± 5.78	35	37	41	31
*x*^2^/t		0.043	0.076	0.091	0.019	0.026	0.076	0.041
*p*		0.872	0.792	0.719	0.37	0.213	0.128	0.464

### 3.2 Comparison of cognitive function between the two groups of patients

**Figure 2:**
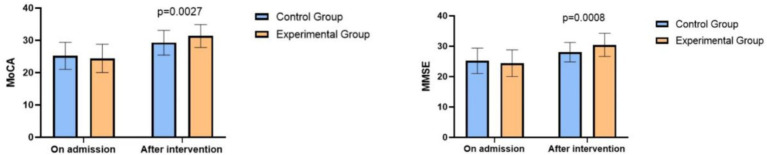
Comparison of MoCA and MMSE Scores between the two groups of patients.

Upon admission, the two groups showed similar MoCA and MMSE scores (*p* > 0.05). After the intervention, patients receiving ward noise management and comprehensive nursing exhibited significantly better recovery in cognitive function than those with conventional care, with a *p*-value of 0.0027 for MoCA and 0.0008 for MMSE, as shown in Figure 2. For MoCA, Cohen’s d was 0.75 (large effect), with a 95% CI of [0.45, 1.05], and for MMSE, Cohen’s d was 0.80 (large effect), with a 95% CI of [0.50, 1.10]. These additional metrics indicate a significant and large effect of the intervention, reinforcing the improvement in cognitive function beyond statistical significance.

### 3.3 Comparison of motor function between the two groups of patients

**Figure 3:**
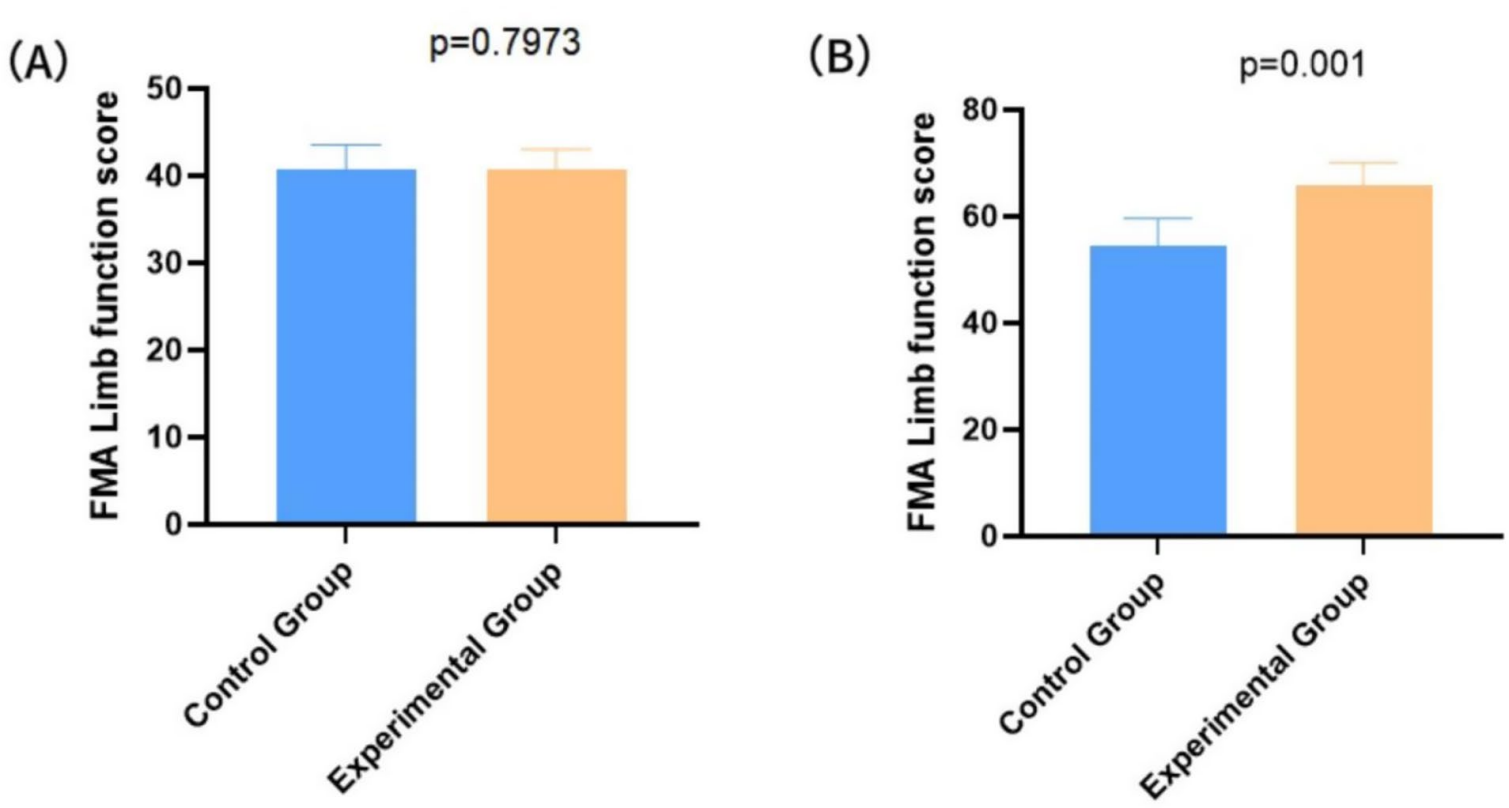
Comparison of limb function levels between the two groups. **(A)** FMA scores of the two groups before intervention; **(B)** FMA scores of the two groups after intervention.

Upon admission, the FMA score for the control group was 41.84 ± 4.67, while the experimental group had an FMA score of 41.08 ± 3.31. No significant difference was observed between the two groups (*p* = 0.7973) (Figure 3A). After the intervention, patients with the combined nursing protocol showed a significantly higher FMA score (67.15 ± 11.86) than those with conventional care (53.44 ± 11.03) (*p* = 0.001), as shown in Figure 3B. For the post-intervention FMA scores, the baseline-adjusted mean difference between groups was 13.71 (95% CI: 9.94 to 17.48), accompanied by a Cohen’s d of 0.75 (95% CI: 0.45 to 1.05), indicating a substantial effect of the combined nursing care protocol.

### 3.4 Comparison of psychological states between the two groups

**Figure 4:**
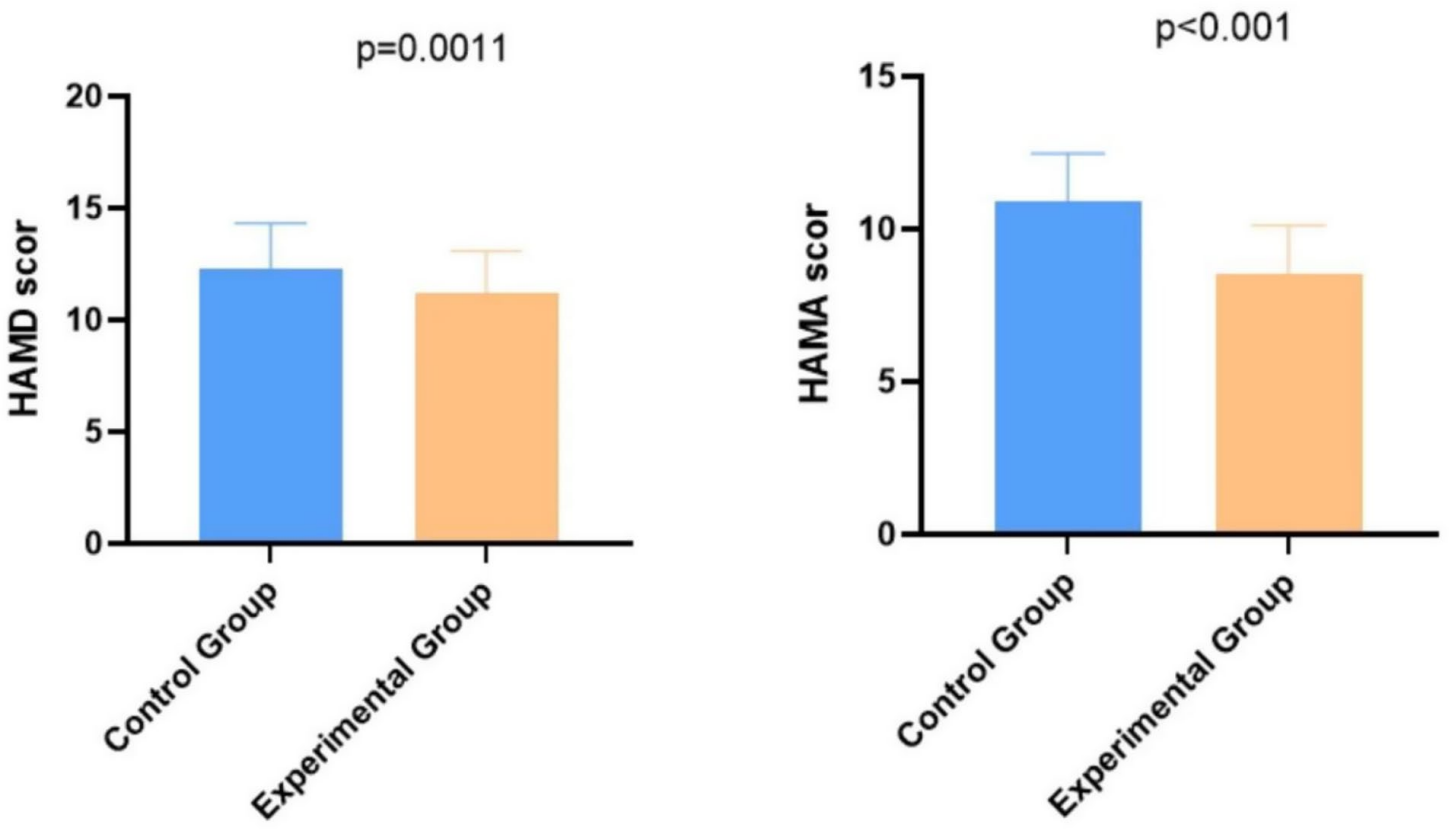
Comparison of psychological states between the two groups.

The HAMD scores were 12.01 ± 2.56 in the control group and 6.98 ± 4.56 in the experimental group, and the HAMA scores were 11.01 ± 3.17 in the control group and 8.19 ± 2.74 in the experimental group. As shown in Figure 4, patients in the experimental group had significantly lower HAMD and HAMA scores than those in the control group, indicating a more stable psychological state in patients after undergoing noise management combined with comprehensive nursing intervention. The *p*-values were 0.0011 for HAMD and <0.001 for HAMA. The mean difference in post-intervention HAMD scores was −5.03 (95% CI: −6.25 to −3.81), and for HAMA, the mean difference was −2.82 (95% CI: −3.80 to −1.84). These corresponded to Cohen’s d values of 1.10 (95% CI: 0.65 to 1.55) and 0.95 (95% CI: 0.58 to 1.32), respectively, reinforcing the significant improvement in psychological health. These additional metrics indicate a significant and large effect of the intervention, reinforcing the improvement in psychological health beyond statistical significance.

### 3.5 Comparison of quality of life between the two groups

As shown in Table 2, the SS-QOL scores of the two groups of patients upon admission were similar (148.73 ± 24.65 vs. 150.09 ± 26.37) (*p* > 0.05). After the intervention, a significantly larger increment of the SS-QOL score was observed in the experimental group (183.24 ± 31.13) than in the control group (170.01 ± 25.66) (*p* = 0.006), indicating that ward noise management integrated with comprehensive nursing care offers a better quality of life for post-stroke patients when compared to routine care.

**Table 2:** Comparison of quality of life between the two groups.

	*n*	Before intervention	After intervention
Control	72	148.73 ± 24.65	170.01 ± 25.66
Experimental	72	150.09 ± 26.37	183.24 ± 31.13
*t*		0.413	3.719
*p*		0.704	0.006

Additionally, effect sizes (Cohen’s d) and 95% confidence intervals (CI) were calculated for key outcomes. The baseline-adjusted mean difference for the SS-QOL score was 13.23 (95% CI: 3.83 to 22.63), with a Cohen’s d of 0.65 (95% CI: 0.30 to 1.00), indicating a moderate to large effect of the intervention on quality of life.

### 3.6 Comparison of complication incidence rate between the two groups

The control group reported 18 cases of cerebral edema, 10 cases of infections, 12 cases of electrolyte imbalances, 25 cases of fever, 7 cases of seizures, and 16 cases of pressure ulcers. The experimental group documented 10 cases of cerebral edema, 4 cases of infections, 8 cases of electrolyte imbalances, 15 cases of fever, 3 cases of seizures, and 12 cases of pressure ulcers. Given that patients could experience more than one complication, the total incidence rate per group is calculated as the number of patients who experienced at least one complication. The control group had a total incidence rate of 68%, while the experimental group had a rate of 43%. Based on the ITT analysis, noise management and comprehensive care significantly reduced the risk of complications compared to conventional care (*p* = 0.021). The OR was 0.35 (95% CI: 0.18 to 0.70), reflecting the intervention’s efficacy even after accounting for potential data loss during the follow-up period. As shown in Table 3.

**Table 3:** Comparison of complication incidence rate between the two groups.

Group	*n*	Cerebral edema	Infections	Electrolyte imbalance	Fever	Seizures	Pressure ulcers	Total incidence rate	*p*
Control	72	18	10	12	25	7	16	68%	0.021
Experimental	72	10	4	8	15	3	12	43%	

Additionally, effect sizes (Cohen’s d) and 95% CI were calculated for key outcomes. For the incidence of complications, the OR was 0.35 (95% CI: 0.18 to 0.70), indicating that the experimental group had significantly lower odds of experiencing complications compared to the control group.

## 4 Discussion

Stroke is a serious neurological disorder that can lead to severe consequences, including cognitive decline, motor impairment, mental health issues, and reduced quality of life. During post-stroke rehabilitation, comprehensive nursing intervention is a widely recognized approach that provides holistic care—encompassing physical rehabilitation, psychological support, medication management, and social support—thereby improving patient prognosis (16).

Studies indicate that effective ward noise management meaningfully contributes to the recovery of cognitive function in post-stroke patients (17–19), likely by improving attention and the quality of cognitive training. In the present study, cognitive function improved significantly in both groups after the intervention, with greater improvement observed in patients receiving the integrated protocol of ward noise management and comprehensive nursing care. This finding aligns with that of Bateman et al. (2016) (20) and provides preliminary evidence that combining noise management with comprehensive nursing may enhance cognitive recovery after stroke.

The neurobiological mechanisms through which noise management may improve MoCA and MMSE scores are likely multifaceted. Excessive ward noise acts as a potent environmental stressor, which can dysregulate stress hormones such as cortisol—known to impair hippocampal function and executive performance. Maintaining a quiet environment, especially at night, promotes better sleep quality and longer periods of slow-wave sleep. This is critical for memory consolidation and glymphatic clearance of neurotoxic waste products from the brain, both essential for neural plasticity and cognitive recovery after stroke. The superior cognitive outcomes in the experimental group suggest that reducing acoustic disruption serves not only as a comfort measure but also as a neuroprotective intervention. Minimizing nocturnal noise helps stabilize the sleep–wake cycle, reduces oxidative stress, and supports prefrontal cortex functions—such as attention, orientation, and memory—assessed by the MMSE and MoCA. Furthermore, prior research has linked reduced ward noise to faster recovery, as patients can focus and engage more effectively in rehabilitation exercises within a calm setting (18, 21).

Comprehensive nursing represents a holistic healthcare model (22). For stroke patients, it integrates multiple aspects of treatment, including physical rehabilitation, psychological support, medication management, and social support (23). Physical rehabilitation plays a central role. Our results indicate that integrating ward noise management with comprehensive nursing more effectively enhances motor function compared to standard care alone. Limb paralysis and motor impairment commonly follow stroke, substantially compromising patients’ quality of life. Tailored exercise plans and rehabilitation training enable patients to progressively regain muscle control and mobility, mitigating the sequelae of paralysis (24).

Psychological support is another key component. Stroke patients frequently experience depression and anxiety (25), which can hinder rehabilitation. One study reported better medication adherence following comprehensive nursing intervention, thereby reducing the risk of cardiovascular events (26). Evidence also suggests that individualized comprehensive care plans promote recovery in both cognitive function and psychological well-being (27, 28). In line with this, our study found that patients receiving combined noise management and comprehensive nursing showed better mental health and quality of life than those under routine care. By providing professional psychological support and emotion-regulation strategies, comprehensive nursing helps patient’s better cope with emotional challenges. Moreover, hospitals or rehabilitation centers implementing comprehensive nursing for stroke patients have demonstrated lower readmission rates, indicating that such care reduces complications and setbacks while improving long-term prognosis (29).

Notably, the incidence of various complications was significantly lower in the experimental group than in the control group. These complications were rigorously validated by independent specialists, and the low attrition rate (5.5% overall) during the one-year follow-up further strengthens the reliability of our prognostic findings. The observed reduction may be explained by noise management alleviating tension and anxiety triggered by high-decibel sounds, while more tailored and holistic care plans help avert scenarios that could negatively affect recovery. This result is consistent with prior research (30, 31) and underscores the clinical importance of integrating such strategies to mitigate complication risks in stroke patients.

Despite these encouraging findings, several limitations should be acknowledged. First, this was a single-center study; multicenter trials are needed to confirm the generalizability of the results. Second, the sample size was relatively small, which may affect the stability of the statistical analysis. Third, the study did not provide detailed descriptions of the specific noise-control methods or the exact components of the comprehensive nursing protocol, which could influence the reproducibility of the outcomes.

Noise in hospital settings remains a significant challenge for stroke recovery. However, through dedicated equipment, environmental design, and staff training, substantial improvements can be achieved—enhancing both the rehabilitation experience and success rates for patients. In summary, the combined application of comprehensive nursing and structured ward noise management appears to be a promising strategy for supporting cognitive function, mental health, and rehabilitation prognosis in stroke patients, though further validation in larger, multicenter trials is warranted.

## 5 Conclusion

This study highlights the critical role of ward noise management and comprehensive nursing intervention in the rehabilitation of stroke patients. Effective noise control was shown to improve sleep quality and support cognitive recovery, while holistic nursing provided multidimensional rehabilitation support, contributing to better psychological well-being and functional outcomes. Within the limitations of this single-center trial, the integrated approach demonstrated favorable effects on cognition, mental health, and overall prognosis in the studied population. Future research should further explore optimized protocols for these interventions to enhance rehabilitation effectiveness and support evidence-based practice in stroke care.

## Data Availability

The raw data supporting the conclusions of this article will be made available by the authors, without undue reservation.
