# Supplementary material for: The impact of ward noise management and comprehensive nursing intervention on cognitive function, psychological health, and prognosis of post-stroke patients: a randomized controlled trial
Source: Front Neurol. 2026 Mar 3;17:1778278. doi: 10.3389/fneur.2026.1778278 (PMC12992026; doi:10.3389/fneur.2026.1778278)
Supplement: Supplementary file 1 [file Presentation_1.PPTX]

## Slide 1
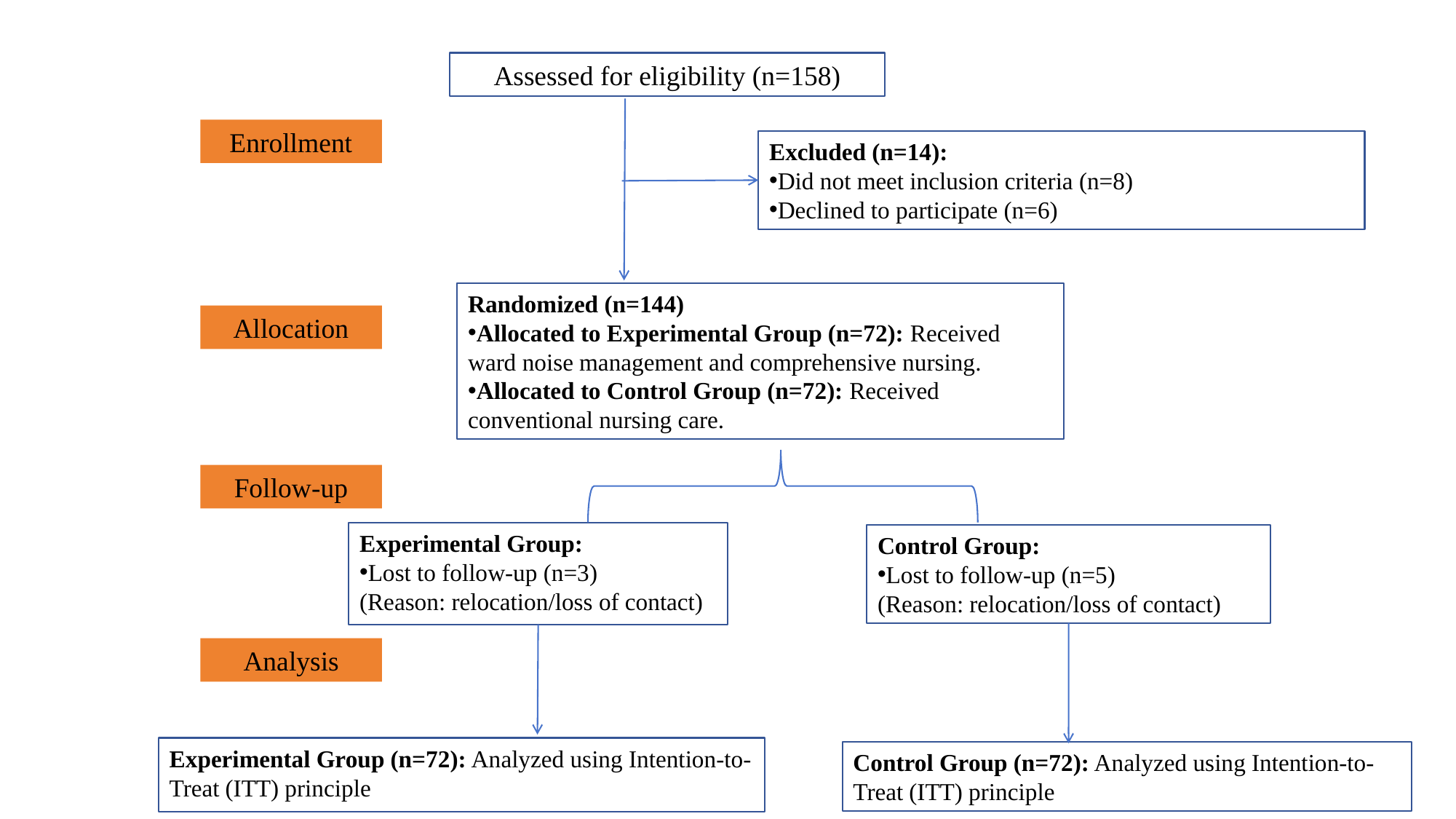

Assessed for eligibility (n=158)
Enrollment
Excluded (n=14):
Did not meet inclusion criteria (n=8)
Declined to participate (n=6)
Randomized (n=144)
Allocated to Experimental Group (n=72): Received ward noise management and comprehensive nursing.
Allocated to Control Group (n=72): Received conventional nursing care.
Allocation
Follow-up
Experimental Group:
Lost to follow-up (n=3)
(Reason: relocation/loss of contact)
Control Group:
Lost to follow-up (n=5)
(Reason: relocation/loss of contact)
Analysis
Experimental Group (n=72): Analyzed using Intention-to-Treat (ITT) principle
Control Group (n=72): Analyzed using Intention-to-Treat (ITT) principle
